# Triage of frail elderly with reduced exercise tolerance in primary care (TREE). a clustered randomized diagnostic study

**DOI:** 10.1186/1471-2458-12-385

**Published:** 2012-05-28

**Authors:** Yvonne van Mourik, Karel GM Moons, Loes CM Bertens, Johannes B Reitsma, Arno W Hoes, Frans H Rutten

**Affiliations:** 1 Julius Center for Health Sciences and Primary care, University Medical Center Utrecht, PO box 85500, Stratenum 6.131, 3508, AB, Utrecht, the Netherlands

**Keywords:** Reduced exercise tolerance, Dyspnoea, Breathlessness, Heart failure, COPD, Frail, Elderly, Screening

## Abstract

**Background:**

Exercise reduced tolerance and breathlessness are common in the elderly and can result in substantial loss in functionality and health related quality of life. Heart failure (HF) and chronic obstructive pulmonary disease (COPD) are common underlying causes, but can be difficult to disentangle due to overlap in symptomatology. In addition, other potential causes such as obesity, anaemia, renal dysfunction and thyroid disorders may be involved.

We aim to assess whether screening of frail elderly with reduced exercise tolerance leads to high detection rates of HF, COPD, or alternative diagnoses, and whether detection of these diseases would result in changes in patient management and increase in both functionality and quality of life.

**Methods/Design:**

A cluster randomized diagnostic trial. Primary care practices are randomized to the diagnostic-treatment strategy (screening) or care as usual.

Patient population: Frail (defined as having three or more chronic or vitality threatening diseases and/or receiving five or more drugs chronically during the last year) community-dwelling persons aged 65 years and older selected from the electronic medical files of the participating general practitioners. Those with reduced exercise tolerance or moderate to severe dyspnoea (≥2 score on the Medical Research Counsel dyspnoea scale) are included in the study.

The diagnostic screening in the intervention group includes history taking, physical examination, electrocardiography, spirometry, blood tests, and echocardiography. Subsequently, participants with new diagnoses will be managed according to clinical guidelines. Participants in the control arm receive care as usual. All participants fill out health status and other relevant questionnaires at baseline and after 6 months of follow-up.

****Discussion**:**

This study will generate information on the yield of screening for previously unrecognized HF, COPD and other chronic diseases in frail elderly with reduced exercise tolerance and/or exercise induced dyspnoea. The cluster randomized comparison will reveal whether this yield will result in subsequent improvements in functional health and/or health related quality of life.

**Trial registration:**

ClinicalTrials.gov NCT01148719

## Background

Reduced exercise tolerance and exercise induced dyspnoea are very common complaints in the elderly, with prevalence rates varying from 20% to 60% [1,2]. In many of the elderly with these complaints heart failure (HF) and/or chronic obstructive pulmonary disease (COPD) may be involved [2]. Multiple causes, however, should be considered, including obesity, anaemia, renal dysfunction and thyroid disorders.

Because of overlap in clinical presentation [3-5], it is difficult to disentangle HF and COPD in the clinical assessment, resulting in both false-negative and false-positive diagnoses of both diseases in primary care [6-10], with subsequent undertreatment and unnecessary drug therapy, respectively. We suspect that especially in the frail, i.e. those prescribed multiple drugs and with multimorbidity, the prevalence of ‘unrecognized’ underlying disease causing reduced exercise is potentially high. For both COPD and HF, but also for other possible underlying diseases such as anaemia and thyroid disorders, effective interventions (i.e. life style interventions and drugs) are available that can improve symptoms, functionality and quality of life and may reduce hospital admissions and mortality [4,11]. Potentially, a substantial beneficial effect in health outcome can be achieved by performing diagnostic tests (screening) in the early course of these diseases.

We designed a cluster randomized trial to examine whether screening frail elderly for HF, COPD, and easy to detect other diseases (such as renal dysfunction, anaemia and thyroid disorders) yields a high proportion of previously unrecognized disease and whether subsequent targeted interventions improves patient outcome. Moreover, we will assess whether such a strategy is cost-effective.

### Key objectives

To determine the yield of screening for previously unrecognized HF, COPD and other chronic diseases (like anaemia, renal dysfunction, thyroid disorders) in frail elderly with reduced exercise tolerance and/or exercise induced dyspnoea.

To assess the effect of the diagnostic screening and subsequent targeted interventions on functional health and health related quality of life after 6 months of follow-up.

To assess the cost-effectiveness of screening and subsequent targeted management.

To identify the most cost-effective combination of screening tests.

## Methods

### Study design

**Figure 1:**
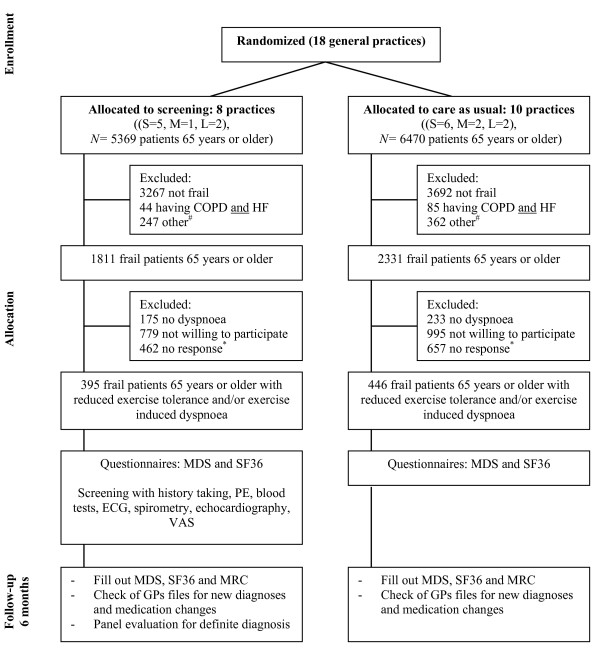
**Study scheme.** S = small (2400 patients), M = medium (4800 patients), L = large (7200 patients), COPD = chronic obstructive pulmonary disease, HF = heart failure, MDS = Minimal Data Set, SF36 = 36 Item Short-Form Health status questionnaire, PE = physical examination, ECG = electrocardiogram, VAS = visual analog scale, MRC = Medical Research Council dyspnoea questionnaire, GP = general practitioner, ^#^other includes cognitive problems (like dementia) and unable to travel to the general practice, ^*^after sending 2 written invitations.

A cluster randomized trial with primary care practices at the unit of randomization, comparing the effects of the screening program (with subsequent targeted interventions in newly detected diseases) compared to usual care on the number of previously unrecognized chronic diseases and on patient outcomes and costs. See Figure 1 for the study scheme.

### Study population and recruitment

Patients aged 65 years or over are selected from the electronic medical file of the participating general practitioners (GPs). All participants are recruited by GPs from the catchment area of the ‘network Utrecht care for elderly (NUZO)’. In the Netherlands all citizens are registered with a GP, irrespective of (co)treatment by a specialist, including patients living in a home for the elderly, but except those living in a nursing home or hospice. Our sample can therefore be considered as a representative sample of community dwelling patients aged 65 year or over.

To identify frail elderly we will use the criteria of the ‘transition project Elderly Care Mid Utrecht (Om-U)’; i.e. men or women who are chronically prescribed five or more different types of drugs during the last year, and/or have three or more chronic or life-threatening diseases.

Patients already known with an established diagnosis of COPD (according to the GOLD-criteria [11]) AND also known with established HF (i.e. diagnosed by a cardiologist, backed-up by an echocardiogram [4]) are excluded. Patients who are unable to travel to the surgery of the GP or with severe cognitive problems are also excluded.

The participating GP sends a letter to all eligible frail patients, including an information letter about the study. Patients willing to participate fill out two short questionnaires concerning dyspnoea and exercise tolerance. Those who scored positive on reduced exercise tolerance or had a ≥2 score on the Medical Research Counsel (MRC) dyspnoea scale [12] are included in the study (see Additional file 1 for an overview of the inclusion criteria).

Written informed consent is obtained at the first appointment before any study procedure is undertaken.

### Randomization

Primary care practices are randomly allocated to either the diagnostic screening with subsequent targeted intervention strategy or care as usual. Randomization is considered necessary because we aim to quantify the clinical effectiveness of the strategy (test results followed by targeted treatment initiated by the GP). Cluster randomization is used to avoid contamination of GPs and participants between the two groups, which can occur if randomization is performed at an individual participant level.

Randomization is performed by a computer-based program, using the minimization method. Minimization is based on the size of the general practice (number of enlisted men and women) and ensures that the two arms are balanced in the number of participants.

### Sample size

We estimated that with our diagnostic strategy at least 15% of the population under study would be diagnosed with newly detected HF and/or COPD. These numbers are based on previous diagnostic studies showing that among elderly with COPD 20% had previously unknown HF [6].

To detect a 10% difference between the 2 arms in diagnoses of HF and/or COPD, using an alpha of 0.05, a power of 0.80 and an intracluster correlation coefficient of 0.05[13], 301 participants per arm are needed. Considering a drop-out of 10% we aim to include 400 participants in each arm (total 800). We calculated that around 35 general practices should participate to recruit 800 participants.

### Study procedures

At the first appointment, all participants fill out a questionnaire of the ‘Dutch National Care for the Elderly Program (ZonMw-NPO)’, i.e. the Minimal Data Set (MDS)[14] on functionality and a questionnaire on general health related quality of life, i.e. the short form-36 (SF-36) [15,16].

Subsequently, participants receive detailed information on the study procedures in the diagnostic screening strategy arm or care as usual arm, depending on the random allocation of their GP practices.

After six months the electronic medical files of all participants are searched to document changes in medication status, new diagnoses and use of care during the past six months and participants are asked to fill out again the MRC dyspnoea, MDS and SF-36 questionnaires.

### Screening strategy and care as usual

The diagnostic screening includes the following tests: standardized questionnaire on medical history, symptoms and current drug use (participants are asked to bring their medication containers), physical examination with special attention for the heart and lungs, blood tests, electrocardiogram (ECG), spirometry with pre- and post-bronchodilator measurements and echocardiography including tissue Doppler imaging.

The physical examination is performed by a trained physician in a standardized manner. Venous blood samples are taken and after centrifugation specimens of plasma and cells will be stored at −70 degrees Celsius. The blood tests include measurements of plasma B-type natriuretic peptide measurements (NT-pro-BNP), glucose levels, creatinin levels (with a calculated estimated glomerular filtration rate (eGFR) according to MDRD (modification of diet in renal disease)), haemoglobin and Thyroid-Stimulating Hormone (TSH) levels. A standard 12-lead ECG is recorded by a trained employee of the GPs’ laboratory (Saltro) and classified according to the Minnesota coding criteria by a single cardiologist, blinded to all other test results [17].

The spirometry is also performed by a trained employee of Saltro before and 20 minutes after administration of ipratropiumbromide inhalation with an inhalation chamber, and subsequently read and interpreted by a pulmonologist.

Echocardiography is performed with a mobile echocardiography (Vivid-i) by a trained and experienced cardiac sonographer from Saltro. All echocardiographic images will be stored and interpreted by a single cardiologist who is blinded to all other data.

After every investigation, the participant fills out a visual analog scale (VAS) to measure the burden experienced during the investigation (0 = not burdening at all, 10 = extremely burdening).

All investigations take place in the patient’s general practice office. Every participant in the diagnostic screening group receives all measurements. About two weeks after the investigations, the GP of the participant in the diagnostic screening arm will receive all results, with (preliminary) diagnoses (presence or absence of HF and/or COPD and of other diseases) including a treatment advice. The GP can then initiates or adjusts the treatment according to current clinical guidelines.

In the care as usual group, the participating GP only receives the answers to the questionnaires about symptoms (answers to the MRC dyspnoea and exercise tolerance questionnaires) from the participants. It is up to the GP to decide whether he/she actively starts any investigations or treatment depending on the answers provided.

### Outcome measures

#### HF and COPD

The presence of HF and/or COPD present or absent will be determined six months after the initial investigations by an outcome panel that will evaluate all diagnostic test results and the effect of treatment during six months follow-up.

For the diagnosis of COPD, airflow obstruction with spirometry is a prerequisite and defined as a post-dilatory ratio of the forced expiratory volume in one second (FEV1) to the forced vital capacity (FVC) <0.70 (FEV1/FVC ratio <0.70). To classify the severity of obstruction, the GOLD-criteria [11] will be applied by the panel.

For the diagnosis of HF, the panel applies the criteria of the European Society for Cardiology (ESC) [4]. HF is considered present when participants have suggestive symptoms (i.e. breathlessness at rest or on exercise, fatigue, tiredness, ankle swelling) and signs (i.e. pulmonary rales, raised jugular venous pressure, peripheral oedema, laterally displaced apical beat) in combination with objective echocardiographic evidence of cardiac dysfunction at rest. In participants who use diuretics for hypertension, signs of volume overload could be masked and therefore in these participants signs of fluid overload are not obligatory. Participants classified as HF by the panel will further be classified as systolic HF, heart failure with preserved ejection fraction (HFPEF) or right-sided HF (cor pulmonale).

For systolic HF, participants need to have a left ventricular ejection fraction (LVEF) ≤45%. To diagnose HFPEF, there must be echocardiographic evidence of diastolic structural or functional abnormalities (abnormal left ventricular relaxation or diastolic stiffness), left atrial enlargement or left ventricular hypertrophy (LVH) in combination with a LVEF >45% [18]. Participants with echocardiographically-determined LVH and an indexed left atrial volume of ≥34 ml/m^2^ or tissue Doppler abnormal mitral inflow or pulmonary venous flow profile are also classified as having diastolic dysfunction. In participants with atrial fibrillation (AF) a complete diastolic assessment is not feasible and therefore we consider an elevated indexed LA volume sufficient to classify diastolic dysfunction in participants with AF. Isolated right-sided HF is defined as increased pulmonary artery pressure (calculated systolic pulmonary artery pressure >40 mmHg) and a LVEF >45%.

The panel also assessed the most likely cause of HF based on the information from the investigations of the diagnostic screening.

#### Other diagnoses

Other new diagnoses considered as an explanation of reduced exercise tolerance or dyspnoea are (persisting) asthma, pulmonary restriction, obesity, anaemia, renal dysfunction, thyroid disorders and atrial fibrillation.

For the diagnosis asthma a combination of symptoms (dyspnoea, wheezing, coughing, sputum production), allergy and/or hyperreactivity is needed, and an increase of >12% in forced expiratory volume in one second (FEV1) from pre- to post-dilatory measurements further confirms the diagnosis. The diagnosis of pulmonary restriction is based on a post-dilatory forced vital capacity (FVC) post-dilatory < 80% of predicted, in the absence of a diagnoses of COPD.

Obesity is defined as a body mass index (BMI) above 30 kg/m^2^.

Anaemia is considered present if Haemoglobin (Hb) is below 7.0 mmol/l in women and below 8.0 mmol/l in men aged 65 to 69 years. In patients aged 70 years or over anaemia is present if Hb is below 6.9 mmol/l in men and below 6.8 mmol/l in women.

Renal dysfunction is considered present if the calculated, based on the MDRD formula, eGFR is below 90 ml/min/1.73 m^2^ for men and 80 ml/min/m^2^ for women. Severity of renal dysfunction is divided into 4 stages; mild (GFR between 60 ml/min/1.73 m^2^ and 80 (women) or 90 (men), moderate (GFR between 30 and 59 ml/min/1.73 m^2^), severe disease (GFR between 15 and 30 ml/min/1.73 m^2^) and kidney failure (GFR below 15 ml/min/1.73 m^2^) [19].

Thyroid disorders are divided into hypo- or hyperthyroidism. When the Thyroid-Stimulating Hormone (TSH) level is below 0.35 or above 5.5 mu/l, Free Thyroxin 4 (FT4) will be measured. Hypothyroid disease is considered present when TSH >5.5 mu/l and FT4 <11 pmol/l. Subclinical hypothyroid disease is when TSH >5.5 mu/l, and FT4 within normal limits (i.e. between 11 and 23 pmol/l). Hyperthyroid disease is considered present when TSH <0.35 mu/l and FT4 >23 pmol/l. Subclinical hyperthyroid disease is a TSH <0.35 mu/l and FT4 within normal limits.

Atrial fibrillation is classified according to the Minnesota coding [17].

#### Functional health

Functional health is measured with the Minimal Data Set (MDS). The dimensions asked in the MDS are: living situation, perceived health and quality of life, health related quality of life (questions of the index EuroQol Five-Dimensional Questionnaire (EQ-5D_index))[20], activities of daily living (ADL) functioning (questions from Katz-15[21]) and health care use (hospital admissions, visits of GP outside office hours, home care and temporally admission to a nursing home).

#### Health related quality of life

Health related quality of life is measured by the SF-36 and the EQ-5D_index (included in the MDS) questionnaires. The SF-36 is divided into nine subscales: physical functioning, social functioning, limitations in usual role activities due to physical problems, limitations in usual role activities due to emotional problems, bodily pain, change in health, general mental health and vitality health change and general health perception. Scores range from 0 and 100.

The EQ-5D_index is a questionnaire with five dimensions (mobility, self-care, usual activities, pain/discomfort and anxiety/depression), which are divided into three degrees of severity, ‘no problem’, ‘some problems’ or ‘major problems’. A single index score can be produced using information from these five dimensions.

Higher scores are associated with a better health related quality of life.

#### Costs

The electronic medical file from the GP is used to collect information about direct costs during the follow-up period of 6 months. Information about GP visits, investigations (i.e. blood tests, ECG, spirometry), outpatient visits, hospitalizations and medication use are collected in both groups. In the diagnostic screening group additionally costs are calculated for all performed test at baseline. Prices will be based on market prices or tariffs.

### Final diagnosis by the panel

The final diagnoses are set by a joint panel consisting of three members, a cardiologist, a pulmonologist and a GP. Consensus diagnosis by the panel will be our reference test for the diagnosis of COPD and HF, in case of any discongruence with the classification described before. This is an established method in case an irreproachable reference standard is lacking, as is the case for HF [22]. In case of no consensus the majority decision will be used. In analogy with earlier studies, the panel will use all available information from the diagnostic and follow-up results: the latter to include the effect of targeted therapy in the decision whether a disease is present. The reproducibility of the panel diagnoses will be evaluated by re-testing a random sample of 10% by the same outcome panel.

The joint panel evaluation is divided in three steps; step 1 history taking, physical examination, blood tests, ECG and spirometry, step 2 adding NT-pro-BNP and step 3 adding echocardiography and follow-up. After step 1 and 2 the panel gives a percentage (0-100%) of their suspicion for the different outcomes. With this, we can evaluate how the panel reaches their final diagnoses (set at step 3) and which investigations add most in reaching this decision.

The above evaluation is also done individually by a GP, a pulmonologist and a cardiologist, but the procedure is changed slightly by dividing step 3 in two steps whereby step 3 includes only echocardiography and at step 4 the follow-up is included and the final diagnoses are made. We will evaluate this individual approach and compare it with the more time consuming joint panel evaluation.

### Blinding

In our single blind study we apply no active measures for blinding the group assignment. The GPs are invited to participate in a study detecting unrecognized HF and COPD. If they agree to participate, their practice is randomized and they receive detailed information. This strategy of selective information is chosen to avoid that GPs in the control group perform all diagnostic tests available in the diagnostic screening program.

Participants will be aware whether their GP practice is allocated to the diagnostic screening arm or to usual care. Researchers are not blinded. There is no risk in observer bias for the outcome, because the questionnaires used to collect outcome measurements are filled out at home by the participants themselves.

### Statistical analyses

The yield of previously unrecognized HF and COPD (and other diseases) will be calculated as a proportion with 95% confidence intervals in both arms of the trial. The absolute difference in yield between the arms will then be calculated together with its 95% confidence interval. We will also calculate how many participants in the diagnostic screening group were initially incorrectly diagnosed with COPD or HF.

The difference in functional health and quality of life between the diagnostic screening and care as usual groups at 6 months will be compared taking into account potential baseline difference of relevant parameters at baseline (ANCOVA), although such differences are expected to be minimal because of the randomization procedure.

Multivariable regression analysis with receiver operating characteristic (ROC) curves and percentages correctly diagnosed participants will be used to analyze which of the test used in the diagnostic screening are most predictive or necessary for setting the diagnosis of HF and COPD in the study group. A multilevel approach is used in the analyses to correct for the fact that we randomized at the GP practice and not at the patient level.

### Cost-effectiveness analysis

The cost-effectiveness of the diagnostic strategy for detection and treatment of (previously unrecognized) HF and COPD (diagnostic screening group) is evaluated and compared with care as usual. Cost-effectiveness is expressed in terms of cost per case of COPD and per case of HF detected, and in terms of cost per Quality Adjusted Life Years gained (Cost-utility analysis). For measuring direct cost, resource quantities are collected prospectively through the electronic medical files from the GPs. Prices are based on market prices or tariffs for the investigations performed under study. Relevant indirect medical costs are taken into account such as participant time invested and travel costs. As the population under study is older than 65 years of age, productivity losses will not be studied. Sensitivity analysis and multivariate uncertainty analysis are performed, conform current Dutch standards for pharmaco-economic research [23].

### Regulation statement

This study is conducted according to the principles of the current version of the declaration of Helsinki and in accordance with the Dutch law on Medical Research Involving Human Subjects Act (WMO).

### Ethics committee approval

The study was approved by the medical ethical committee (METC) of the University Medical Center Utrecht (UMCU), the Netherlands.

## Results of inclusion

Eighteen general practices are included in the study, 8 were randomized to the diagnostic screening intervention and 10 to the care as usual group.

Of the 11.839 patients aged 65 years or older (16% of all registered citizens) enlist with these practices, 35% met the criteria of frailty. The mean age of the 4142 selected frail patients is 75.6 (SD ±6.9) years and 47% is male. In Table 1 patient characteristics, median number of co-morbidities and medication is given for all selected patients. Of the invited frail patients, 73% responded to our invitation and 30% (1249) was willing to participate. Of those frail patients willing to participate, 33% had no complaints of reduced exercise tolerance or dyspnoea (MRC < 2) and therefore are excluded from the study. Patients excluded because they had no complaints were younger (*p* = 0.04) and more often male (*p* = <0.001) as compared to included patients. Eligible patients who refused to participate (*n* = 3301) were slightly older (*p* = <0.001), but they were comparable on gender (*p* = 0.09) compared to participants.

**Table 1:** Characteristics of all invited frail patients (responders and non-responders)

	**All frail patients**^**#**^	**Patients willing to participate**		**Not willing**^**^**^	**No response**^**>**^
		(*n* = 1249)			
		Dyspnoea^*^	**No dyspnoea**^**<**^		
	(*n* = 4142)				
		(*n* = 841)	(*n* = 408)	(*n* = 1774)	(*n* = 1119)
Age (years), mean (sd)	75.6 ± 6.9	74.6 ± 6.3^·^	73.8 ± 6.1^*‡*^	76.3 ± 7.0^*‡*^	75.9 ± 7.2^*‡*^
Male sex, *n* (%)	1956 (47.2%)	419 (49.8%)^†^	270 (66.2%)^*‡*^	763 (43.0%)^*‡*^	504 (45.0%)^*‡*^
Co-morbidities (number), median (IQR)	3 (3–4)	3 (3–4)	3 (3–4)	3 (3–4)	3 (3–4)
Drugs (number), median (IQR)	5 (4–7)	6 (4–7)	5 (3–6)	5 (4–7)	6 (4–7)

^#^All selected frail patients aged 65 years or over, ^*^patients who were willing to participate and fully met the inclusion criteria (MRC dyspnoea ≥2 and/or a positive answer on questionnaire about reduce exercise tolerance), ^<^patients who were willing to participate, but were excluded based on complaints (MRC dyspnoea score ≤1 and only negative answers on the questionnaire about reduce exercise tolerance), ^^^patients who were not willing to participate, ^>^patients who gave no response after sending 2 written invitations. ^*‡*^*P* = <0.05 compared to the patients with dyspnoea, ^†^*p* = ns dyspnoea patients compared to patients not participating in the study (no dyspnoea, not willing and no response), ^·^*p* = <0.05 dyspnoea compared to patients not participating in the study.

Finally, 841 frail patients with complaints of dyspnoea or reduced exercise tolerance were included in the study: 24% with dyspnoea, 16% with reduced exercise tolerance and 60% with both symptoms. The mean age of the participants was 74.6 (SD ±6.3) years and 50% were male. Important co-morbidities were hypertension (70%), diabetes mellitus (31%), ischemic heart disease (31%), hypercholesterolemia (28%), osteo-arthritis (25%) and visual impairment (25%). The most frequently used drugs are related to these co-morbidities. All baseline characteristics of the included patients are described in Table 2.

**Table 2:** Baseline characteristics

	**All**	**Diagnostic screening**	**Care as usual**
	(*n* = 841)	(*n* = 395)	(*n* = 446)
Mean age in years ± sd	74.6 ± 6.3	75.4 ± 6.2	73.8 ± 6.3
Male sex, *n* (%)	418 (49.7%)	176 (44.6%)	242 (54.3%)
*Complaints*			
MRC Dyspnoea score, median (IQR)	2 (2–3)	2 (2–3)	2 (2–3)
Reduced exercise tolerance questionnaire score, median (IQR)	2 (1–3)	2 (0–3)	2 (1–3)
*Cardiovascular co-morbidities*			
Ischemic heart disease, *n* (%)	259 (30.8%)	127 (32.2%)	132 (29.6%)
Heart failure, *n* (%)	42 (5.0%)	22 (5.6%)	20 (4.5%)
Valvular disorders, *n* (%)	59 (7.0%)	24 (6.1%)	35 (7.8%)
Cardiac rhythm disorders, *n* (%)	116 (13.8%)	55 (13.9%)	61 (13.7%)
Hypertension, *n* (%)	591 (70.3%)	292 (73.9%)	299 (67.0%)
Hypercholesterolemia, *n* (%)	234 (27.8%)	94 (23.8%)	140 (31.4%)
Diabetes Mellitus, *n* (%)	262 (31.2%)	130 (32.9%)	132 (29.6%)
CVA or TIA, *n* (%)	99 (11.8%)	44 (11.1%)	55 (12.3%)
*Non-cardiovascular co-morbidities*			
Visual impairment, *n* (%)	207 (24.6%)	98 (24.8%)	109 (24.4%)
Hearing impairment, *n* (%)	93 (11.1%)	43 (10.9%)	50 (11.2%)
COPD, *n* (%)	134 (15.9%)	68 (17.2%)	66 (14.8%)
Asthma, *n* (%)	78 (9.3%)	36 (9.1%)	42 (9.4%)
Mood disorders, *n* (%)	27 (3.2%)	11 (2.8%)	16 (3.6%)
Urinary tract problems, *n* (%)	90 (10.7%)	35 (8.9%)	55 (12.3%)
Osteoporosis, *n* (%)	58 (6.9%)	25 (6.3%)	33 (7.4%)
Malignancies, *n* (%)	51 (6.1%)	18 (4.6%)	33 (7.4%)
Anaemia, *n* (%)	14 (1.7%)	9 (2.3%)	5 (1.1%)
Renal insufficiency, *n* (%)	47 (5.6%)	12 (3.0%)	35 (7.8%)
Thyroid dysfunction, *n* (%)	63 (7.5%)	28 (7.1%)	35 (7.8%)
Osteoarthritis, *n* (%)	212 (25.2%)	126 (31.9%)	86 (19.3%)
Co-morbidities (number), median (IQR)	3 (3–4)	4 (3–4)	3 (3–5)
*Drugs*			
Diuretics, *n* (%)	346 (41.1%)	169 (42.8%)	177 (39.7%)
ACE-i/ARBs, *n* (%)	478 (56.8%)	220 (55.7%)	258 (57.8%)
ß-blockers, *n* (%)	397 (47.2%)	185 (46.8%)	212 (47.5%)
Digitalis, *n* (%)	20 (2.4%)	12 (3.0%)	8 (1.8%)
Oral anticoagulants, *n* (%)	117 (13.9%)	49 (12.4%)	68 (15.2%)
Platelet antagonists, *n* (%)	402 (47.8%)	192 (48.6%)	210 (47.1%)
Treatment for hypercholesterolemia	488 (58.0%)	209 (52.9%)	279 (62.6%)
Anti-diabetic drugs, *n* (%)	223 (26.5%)	109 (27.6)	114 (25.6%)
Treatment for COPD or Asthma, *n* (%)	204 (24.3%)	91 (23.0%)	113 (25.3%)
Drugs (number), median (IQR)	6 (4–7)	5 (4–7)	6 (4–7)

baseline characteristics at selection based on electronic medical fileI Ischemic heart disease includes prior myocardial infarction, angina pectoris, coronary artery bypass grafting, and percutaneous coronary intervention; visual impairment includes cataract, blindness or glaucoma, ACE-i = angiotensin converting enzyme-inhibitor, ARBs = angiotensin receptor blockers.

In total 395 participants were included in the diagnostic screening group and 446 in the care as usual group. The distribution of co-morbidities and drug use of the two arms are shown in Table 2.

## **Discussion**

The screening arm of the trial will generate data on the proportion of frail elderly with reduced exercise tolerance and/or exercise induced dyspnoea that have unrecognized HF, COPD and other chronic diseases (like anaemia, renal dysfunction, thyroid disorders). The comparison of the screened versus usual care arm within this clustered randomized trial will reveal whether screening and subsequent targeted interventions would be beneficial for frail elderly with respect to functional health and/or health related quality of life after 6 months of follow-up. The cost-effectiveness will also be examined.

## Abbreviations

HF, Heart failure; COPD, Chronic obstructive pulmonary disease; GP, General practitioner; MRC dyspnoea, Medical Research Counsel dyspnoea scale; MDS, Minimal data set; SF-36, Short form-36; ECG, Electrocardiogram; NT-pro-BNP, Amino-terminal pro-B-type natriuretic peptide; VAS, Visual analog scale; HFPEF, Heart failure with preserved ejection fraction; LVH, Left ventricular hypertrophy; AF, Atrial fibrillation; Hb, Haemoglobin; MDRD, Modification of diet in renal disease; EGFR, Estimated glomerular filtration rate; TSH, Thyroid-stimulating hormone; FT4, Free thyroxin 4; EQ-5D_index, Index EuroQol Five-Dimensional Questionnaire.

## Competing interests

All authors declare no competing interests.

## Authors’ contributions

KM, AW and FH designed the study. FH coordinated the study. YM and LB managed the study and data collection. YM researched the data, conducted the analyses and wrote the manuscript. All authors read and approved the final draft of the manuscript.

## Pre-publication history

The pre-publication history for this paper can be accessed here:

http://www.biomedcentral.com/1471-2458/12/385/prepub

## Supplementary Material

Additional file 1 Inclusion criteria. Click here for file
