# Supplementary material for: Triage of frail elderly with reduced exercise tolerance in primary care (TREE). a clustered randomized diagnostic study
Source: BMC Public Health. 2012 May 28;12:385. doi: 10.1186/1471-2458-12-385 (PMC3407748; doi:10.1186/1471-2458-12-385)
Supplement: Additional file 1 — Inclusion criteria. [file 1471-2458-12-385-S1.doc]

| • **Age:** **-** 65 years or over **And** | | |
| --- | --- | --- |
| • **Frail:** **-** use of 5 or more different types of medical drugs chronically in the last year and/or **-** having 3 or more chronic or vitality threatening diseases | | |
| **And** • **Medical Research Counsel (MRC) dyspnoea scale grade ≥2 and/or one positive answer on reduced exercise tolerance** *MRC dyspnoea scale^#^* 0. no breathlessness 1. Not troubled by breathlessness except on strenuous exercise. 2. Short of breath when hurrying on the level or walking up a slight hill. 3. Walks slower than most people on the level, stops after a mile or so, or stops after 15 minutes walking at own pace. 4. Stops for breath after walking about 100 yards or after a few minutes on level ground. 5. Too breathless to leave the house or breathless when dressing or undressing. | | |
| *Questionnaire about reduced exercise tolerance** - Do you have a reduced exercise tolerance compared to people of the same age? - Do you feel you’re more tired than people of the same age? - Do you feel you need more recovery after exercise than people of the same age? - Do you have during activities, such as walking, gardening or housework, that you feel your heart pounding or feel palpitations or feel agitated? | Yes □ □ □ □ | No □ □ □ □ |

**Additional file 1 Inclusion criteria**

**^#^** Adapted from Fletcher CM, Elmes PC, Fairbairn MB et al. The significance of respiratory symptoms and the diagnosis of chronic bronchitis in a working population. British Medical Journal 1959;2:257–66, ***** Questions translated, original in Dutch
